# ^99m^Tc-MIBI Scintigraphy for the Preoperative Assessment of Histological Response to Neoadjuvant Chemotherapy in Patients With Osteosarcoma: A Systematic Review and a Bivariate Meta-Analysis

**DOI:** 10.3389/fonc.2020.00762

**Published:** 2020-05-22

**Authors:** Fashuai Wu, Yu Huang, Xin Huang, Silang Fang, Xiaohui Huang, Xin Huang, Zhicai Zhang, Zengwu Shao

**Affiliations:** ^1^Department of Orthopaedics, Tongji Medical College, Union Hospital, Huazhong University of Science and Technology, Wuhan, China; ^2^Department of Otorhinolaryngology, The Third Hospital of Wuhan City, Wuhan, China; ^3^Department of Orthopaedics, The Qichun People's Hospital, Qichun, China; ^4^Department of Orthopaedics, Tongji Medical College, Union Jiangbei Hospital, Huazhong University of Science and Technology, Wuhan, China

**Keywords:** ^99m^Tc-MIBI scintigraphy, uptake change ratios, washout rate, chemotherapy response, osteosarcoma, meta-analysis

## Abstract

**Purpose:** There have been many attempts to preoperatively evaluate the chemotherapy response of osteosarcoma patients using ^99m^Tc-MIBI scintigraphy. However, the evaluations were lacking in consistency. We performed this systematic review and meta-analysis to systematically evaluate the ability of ^99m^Tc-MIBI scintigraphy in preoperatively assessing the response of osteosarcoma patients to neoadjuvant chemotherapy.

**Methods:** For this systematic review and meta-analysis, PubMed, Web of Science, OVID, the Cochrane Library, and CNKI were searched. Eligible studies were included based on the defined criteria. The index test was ^99m^Tc-MIBI scintigraphy, the reference standard was tumor necrosis rate. Quality Assessment of Diagnostic Accuracy Studies-2 was adopted for quality assessment of included studies. The statistical pooling analysis, meta-regression analysis, subgroup analysis, sensitivity analysis, and publication bias of our research were performed using STATA 15.

**Results:** Eight articles with 189 osteosarcoma patients were included in this systematic review and meta-analysis. Our results demonstrated that the threshold effect of our meta-analysis was significant. The uptake change ratio of ^99m^Tc-MIBI scintigraphy had a pooled sensitivity, specificity, positive and negative likelihood ratio, diagnostic odds ratio, and the area under curve of 0.98 (0.58–1.00), 0.68 (0.47–0.84), 3.1 (1.7–5.5), 0.03 (0.00–0.90), 103 (4–3,003), and 0.91 (0.88–0.93) in preoperative assessment of response of osteosarcoma patients to neoadjuvant chemotherapy. Meta-regression analysis and subgroup analysis indicated the factors of method and cut off value may introduce the heterogeneity. The pooled sensitivity, specificity, positive and negative likelihood ratio, diagnostic odds ratio, and the area under curve of washout rate of ^99m^Tc-MIBI were 0.87 (0.69–0.95), 0.91 (0.75–0.97), 9.3 (3.2–27.0), 0.15 (0.06–0.37), 64 (14–301), and 0.89 (0.86–0.92), respectively. Sensitivity analysis and publication bias demonstrated our meta-analysis was reliable.

**Conclusion:** Both the ΔUR and WR derived from ^99m^Tc-MIBI scintigraphy were valuable in preoperatively assessing the response of osteosarcoma patients to neoadjuvant chemotherapy, and ΔUR may possess a more outstanding diagnostic accuracy than WR.

## Introduction

Osteosarcoma is the most commonly seen primary malignant bone tumor that originates from mesenchymal tissue. It usually occurs in the metaphysis of long bones, especially around the knee, of adolescents and young adults (1). It is characterized by its high degree of malignancy, namely high aggressive ability, and early systematic metastasis; 80% of osteosarcoma patients are present with imaging-detectable lung metastatic disease or micro-metastatic disease at their first diagnosis, besides, many patients without metastasis at diagnosis will progress to metastasis during their stages of treatment, and 80% of metastatic lesions arise in the lungs (2, 3). Over past decades, the modality of combining neoadjuvant chemotherapy, surgical resection, and postoperative chemotherapy has been clinically regarded as the standard treatment modality for osteosarcoma patients, and it has remarkably ameliorated patients' survival rate and avoided the necessity of amputation (4, 5). The 5-year survival rate of osteosarcoma patients was around 20–30% in the past with surgery alone, whilst after the advent of multiagent chemotherapy regimens, the long-term survival rate has improved to 65–70% for patients with localized osteosarcoma (2, 6). However, the survival for osteosarcoma has reached a plateau in the last 30–40 years, and patients with tumor recurrence after primary tumor resection or lung metastases continued to have very poor outcomes, which may closely relate to their poor response to chemotherapy (7–9). Thus, more effective therapeutic strategies and helpful and convenient prognostic evaluation methods are highly needed for patients with osteosarcoma.

Response to chemotherapy has been regarded as the most important prognostic factor for osteosarcoma patients (10). Currently, the standard of chemotherapy response assessment is to histologically assess the necrosis rate of the resected tumor after neoadjuvant chemotherapy (11). Osteosarcoma patients with a necrosis rate ≥90% had been defined as good responders, as an indication that they had received an effective preoperative treatment and that might have led to a good outcome (12, 13). The tumor necrosis rate (TNR) also was clinically used to identify patients with a high risk for metastasis and recurrence. However, TNR of the resected lesions is available only postoperatively. Furthermore, this method is complicated and cannot be timely monitoring the tumor response to chemotherapy and applied in non-surgical patients. Using a more convenient method at an early point preferably before treatment or operation to assess whether preoperative chemotherapy was effective was of great significance for the selection and modification of the therapeutic strategy and the evaluation of prognosis. The treatment strategy of suspected poor responders could be intensified and modified earlier, potentially ameliorating the effect of treatment and their prognosis.

There have been many attempts to evaluate the chemotherapy response of osteosarcoma patients before treatment or surgical resection, by various imaging techniques including 18F-FDG PET/CT, MRI, ultrasound, angiography, and radionuclide imaging (14–16). Tc-99mhexakis-2-methoxyisobutylisonitrile (^99m^Tc-MIBI), commonly used as a myocardial perfusion imaging agent, has also been used for assessing the chemotherapy response with high sensitivity and specificity in types of cancers, such as breast cancer, lung cancer, and osteosarcoma (17–20). As far as we know, the evaluation of chemotherapy response can be achieved by analysis of the uptake change ratio (ΔUR) of ^99m^Tc-MIBI in osteosarcoma before and after chemotherapy (21). In addition, the washout rate (WR) of ^99m^Tc-MIBI before chemotherapy, which correlates with the level of P-glycoprotein (P-gp) and multidrug resistance-associated protein (MRP), also has the power to evaluate the chemotherapeutic effect of osteosarcoma patients (22, 23). However, the evaluations were limited in sample size and inconsistent outcome, making the results of low reliability. The aim of this systematic review and meta-analysis was to collect all relevant studies through searching electronic databases to systematically evaluate the performance of ^99m^Tc-MIBI scintigraphy in preoperatively assessing the response of osteosarcoma patients to neoadjuvant chemotherapy.

## Materials and Methods

This systematic review and bivariate meta-analysis was performed and organized according to the Preferred Reporting Items for Systematic Reviews and Meta-Analyses–Diagnostic Test Accuracy, namely the PRISMA-DTA Statement (24).

### Search Strategy

We systematically searched the up-to-date electronic databases which included PubMed, Web of Science, OVID, the Cochrane Library, and CNKI to obtain the articles of using ΔUR or WR of ^99m^Tc-MIBI scintigraphy to preoperatively predict the response of osteosarcoma patients to neoadjuvant chemotherapy. We limited the literature search language to English in the databases of PubMed, Web of Science, OVID, and the Cochrane Library, and to Chinese in CNKI. The last search date was December 20, 2019. To avoid article omission and promote search sensitivity, both MeSH terms and free words were used in our research strategy, which included the terms “^99m^Tc-MIBI” and “Osteosarcoma.” In CNKI, the search strategy was correspondingly translated into Chinese. We also checked and screened the reference lists of all identified primary studies and topic relevant reviews for other potential publications to avoid omitting any potential original articles.

### Inclusion and Exclusion Criteria

An article was considered eligible for inclusion in the systematic review and meta-analysis if it met the following inclusion criteria: (1) It was an original research article on osteosarcoma patients, who could be divided into good responders and poor responders by the TNR of more than or <90% according to Salzer-Kuntschik's grading systems (25). (2) Both ^99m^Tc-MIBI scintigraphy and TNR were used to evaluate the response of osteosarcoma patients to neoadjuvant chemotherapy. (3) Availability of data including ΔUR or WR of ^99m^Tc-MIBI scintigraphy and TNR, which can be obtained directly from the study or indirectly from the curves. (4) Availability of constructing a 2 ^*^ 2 contingency table which contained cases of true and false positives and negatives for performing the meta-analysis with 95% confidence interval (95% CI). However, article types of expert opinions, meeting papers, case reports, editorials, reviews, systematic reviews, and research studies unavailability of useful data or full-text were all excluded. If there were repeated researches, or relevant data was contained and reported in a previous study, we chose the most complete study or the most recent one. All the potential studies were carefully identified and assessed by two independent researchers (F.W. and X.H.) and the decision to include or exclude a study was initially made basing on the study title and abstract, and then the whole article. If met with discrepancies and controversies, decisions were made upon group discussion.

### Data Extraction and Quality Assessment

The two researchers (F.W. and Y.H.) independently performed the data extraction process from eligible studies. To rule out discrepancy, extracted data was then cross-checked by the two researchers, and consensus was reached for all items through discussion. The following items of each eligible study were collected, which included first author, year of publication, countries, sample size, gender, age, study design, methods of retrieving data, dose of ^99m^Tc-MIBI, region of interest, scan time, interested index, criteria to define cut off value, data of cases of true positive, false positive, false negative and true negative, and so on. In the circumstance that data was not presented directly in the publication, we utilized the Engauge Digitizer version 9.8 to obtain useful data from the curves. Quality Assessment of Diagnostic Accuracy Studies-2 (QUADAS-2) tool was implemented in RevMan 5.3 to assess the quality of eligible studies, which was independently performed by two researchers (F.W. and Y.H.) (26). Discrepancies were resolved through group discussion. QUADAS-2 contained four domains: Patient Selection, Index Test, Reference Standard, and Flow and Timing. All four domains were assessed in terms of risk of bias; the first three domains were also assessed in terms of concerns regarding applicability.

### Statistical Analysis

The statistical analysis of our research was performed using STATA 15 (Stata Corporation, College Station, TX, USA). Midas module used an exact binomial rendition for synthesis of the diagnostic test data (27). Pooled sensitivity, pooled specificity, the corresponding positive likelihood (PLR), negative likelihood (NLR), and pooled diagnostic odds ratio (DOR) were calculated. The summary receiver operating characteristic (SROC) curve was constructed. Chi-square (χ^2^) based Cochran Q test and Higgins *I*^2^ statistic test were performed to evaluate the between-study heterogeneity. In our analysis, a *P*-value of <0.05 or an *I*^2^ value of >50% would be regarded as significant heterogeneity. The value of proportion of heterogeneity likely due to threshold effect provided information about the influence of a diagnostic threshold effect on heterogeneity. To detect other potential source of heterogeneity, meta-regression analysis, subgroup analysis, and sensitivity analysis were performed in our analysis. Graphical model of sensitivity analysis consisted of four plots: One, quantile plot of residual-based goodness-of fit. Two, chi-squared probability plot of squared Mahalanobis distances for assessment of the bivariate normality assumption. Three, spikeplot for checking for particularly influential observations using Cook's distance. And four, the scatterplot for checking for outliers using standardized predicted random effects (standardized level-2 residuals). To quantitatively evaluate the publication bias, Deeks' Funnel Plot Asymmetry Test was applied. A *P* < 0.05 hinted a statistically significant publication bias. Fagan nomogram was created to provide information about the likelihood that an osteosarcoma patient with a positive or negative ^99m^Tc-MIBI scintigraphy result was a good responder or not.

## Results

### Search Results

**Figure 1:**
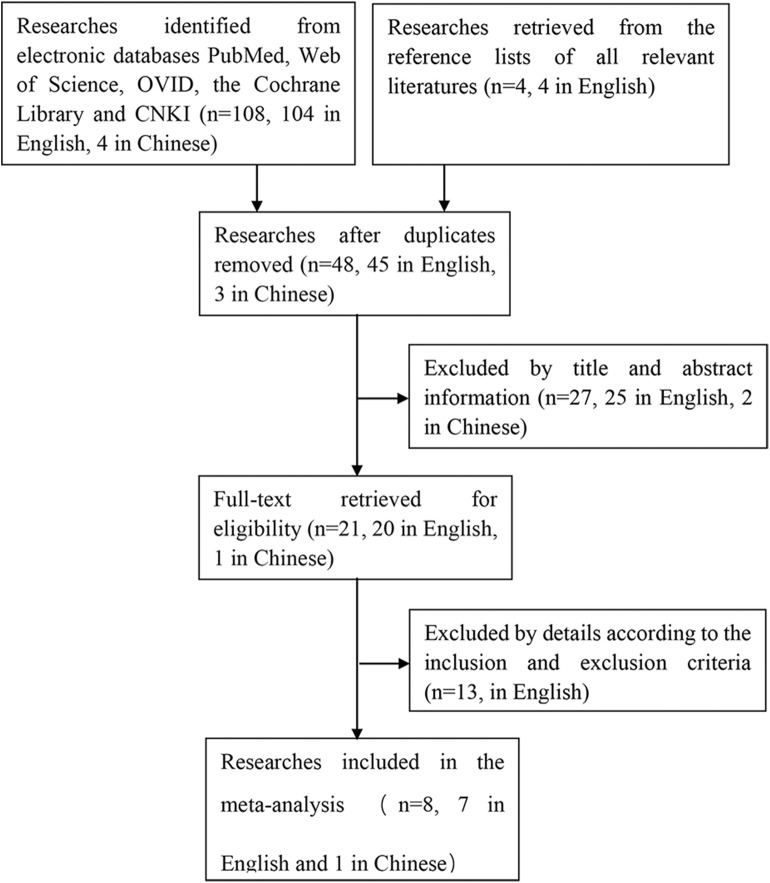
Flow diagram of the study selection process.

The process of searching and retrieving articles for this analysis was shown in Figure 1. A total of 108 potential articles (104 in English and 4 in Chinese) were identified from the electronic databases through our search process. We also screened the reference lists of all relevant literatures to identify other potential publications relating to the topic (*n* = 4, in English). After duplicates were eliminated, there were 48 articles (45 in English, 3 in Chinese) left, of which 27 articles (25 in English, 2 in Chinese) were excluded based on the title and abstract. Thirteen articles in English were excluded after reviewing the full article for the reasons as follows: 1 was eliminated because of full-text unavailable, 8 studies were eliminated for unavailability of getting the cases of true and false positives and negatives to construct a 2 ^*^ 2 contingency table, 2 articles were meeting papers, 2 articles were repeated researches, of which data was reported in previous studies. In the end, 8 articles (7 in English, 1 in Chinese) with a total of 189 osteosarcoma patients fulfilled all the eligible criteria and were evaluated in this meta-analysis (20, 23, 28–33).

### Study Selection and Characteristics

Table 1 shows the main characteristics of all the included studies, among which 6 studies with a total of 148 osteosarcoma patients were about the ΔUR of ^99m^Tc-MIBI between pre- and post-chemotherapy and the histological response of osteosarcoma patients to neoadjuvant chemotherapy. Four studies with 96 osteosarcoma patients were about the WR of ^99m^Tc-MIBI before chemotherapy and the response of osteosarcoma patients to neoadjuvant chemotherapy. The publication year of these eligible studies was from 1997 to 2019. Data of cases of true and false positives and negatives was obtained directly from 6 studies. In the other studies, it was obtained indirectly from the curves by the Engauge Digitizer. The number of patients in every study varied from 12 to 42. The optimal cut-off value was identified for WR to minimize the number of false results. So was ΔUR in 2 studies. In the other 4 studies, osteosarcoma patients with ΔUR of ^99m^Tc-MIBI ≥30% were defined as positive responders (29). ΔUR of ^99m^Tc-MIBI was received by comparing the activities of the osteosarcoma site before and after the chemotherapy in 4 studies or after 3 courses of chemotherapy in 2 studies. WR was measured on pre-chemotherapy ^99m^Tc-MIBI imaging. All original studies of this meta-analysis fulfilled six or more “low risk” answers in the QUADAS-2 tool for methodological quality except for Shinji Miwa's research (Supplementary Figure 1). There was no “high risk” response to any domains. In the domain of applicability concerns, there were all “low risk” responses. In several researches, authors did not mention the sampling methods or inappropriate exclusions in the patient selection part.

**Table 1:** Main characteristics of the studies included in this meta-analysis.

**References**	**Country**	**Number (M/F)**	**Age (y)**	**Study design**	Method*	**^**99m**^Tc-MIBI**	**Cut off value ^**#**^**	**TP, FP, FN, TN**	**ROI**	**Scan time**	**Index**
Soderlund et al. (28)	Sweden	12 (8/4)	Mean: 20	Prospective	1	Adult: 250 MBq, children: reduced by body weight	1	8, 3, 0, 1	Around the tumor	Before and after chemotherapy	ΔUR
Wu et al. (29)	China	30 (26/4)	Mean: 15.1	Retrospective	1/1	Adult: 740 MBq, Children: 250 μCi/kg	2/1	11, 1, 1, 17/11, 0, 1, 18	Around the tumor	Before and after chemotherapy	ΔUR/WR
Miwa et al. (30)	Japan	42	Mean: 22.8	ND	1	600–700 MBq	1	17, 3, 6, 16	On the lesion	Before and after 3 courses of chemotherapy.	ΔUR
Huang et al. (31)	China	22 (15/7)	Median: 16	ND	2	925 MBq (25 mCi)	1	6, 8, 0, 8	ND	Before and after 3 courses of chemotherapy	ΔUR
Burak et al. (32)	Turkey	24 (17/7)	Mean: 25.33	ND	1	370–720 MBq	1	4, 1, 2, 17	On the lesion	Before chemotherapy	WR
Gharehdagh et al. (23)	Iran	25 (12/13)	Mean: 20.48	Prospective	2/2	600–740 MBq	1/1	9, 8, 0, 8/8, 4, 1, 12	Around the lesion	Before and after chemotherapy	ΔUR/WR
Wakabayashi et al. (33)	Japan	17 (11/6)	Mean: 20	Retrospective	1	600–740 MBq	2	9, 2, 0, 6	On the area of increased activity	Before and after chemotherapy	ΔUR
Sohaib et al. (20)	Pakistan	17	ND	Retrospective	1	500 MBq	1	3, 2, 0, 12	Over the lesion	Before chemotherapy	WR

* *1 denoted as data obtained directly from the study; 2 denoted as data obtained indirectly from the curves by the Engauge Digitizer; ^#^1 denoted as the cut-off value was identified to minimize the number of false results; 2 denoted as the cut-off value of ΔUR was defined with the standard of ^99m^Tc-MIBI ≥ 30%; TP, true-positive; FP, false-positive; FN, false-negative; TN, true-negative; ΔUR, the uptake change ratio; WR, washout rate*.

### Meta-Analysis of the Alteration Ratio of ^99m^Tc-MIBI for the Preoperative Assessment of Histological Response to Neoadjuvant Chemotherapy in Osteosarcoma Patients

**Figure 2:**
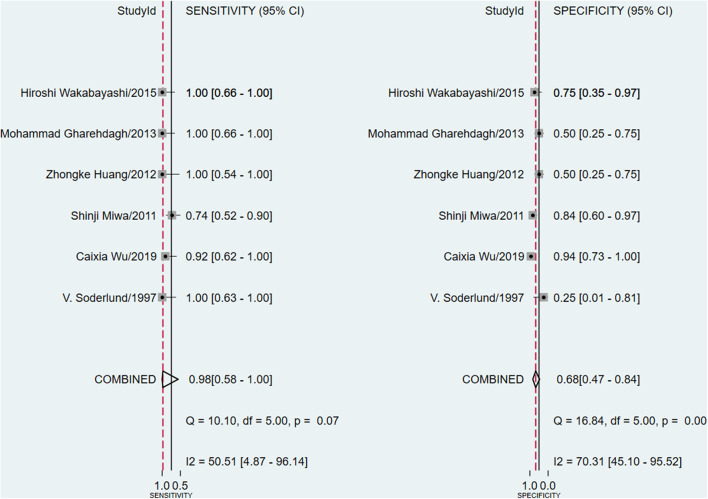
Forest plots of the pooled sensitivity and specificity for the uptake change ratio of ^99m^Tc-MIBI scintigraphy in preoperatively assessing the response of osteosarcoma patients to neoadjuvant chemotherapy.

**Figure 3:**
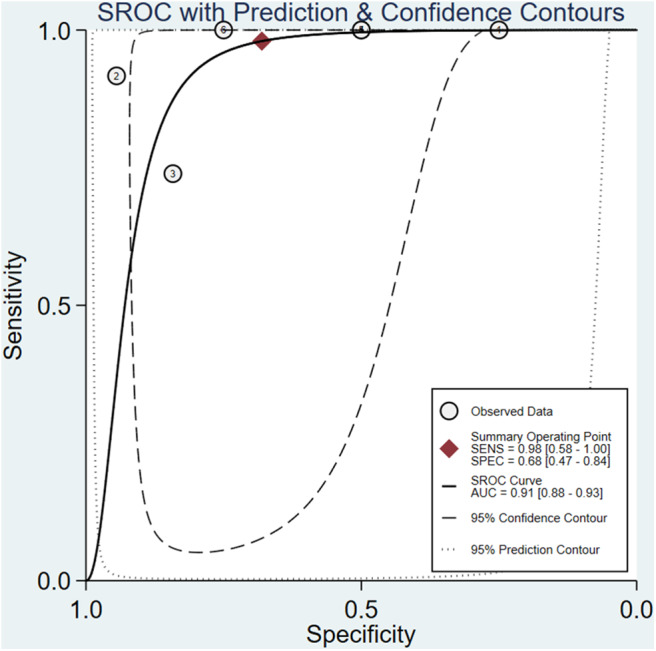
Summary receiver operating characteristic plot for the uptake change ratio of ^99m^Tc-MIBI scintigraphy in preoperatively assessing the response of osteosarcoma patients to neoadjuvant chemotherapy.

The meta-analysis of the ΔUR of ^99m^Tc-MIBI for the preoperative assessment of the histological response of osteosarcoma patients to neoadjuvant chemotherapy included 6 studies with a total of 148 patients. Overall, the pooled sensitivity and pooled specificity were 0.98 (95% CI 0.58–1.00, *I*^2^ = 50.51%, *P* =0.07), and 0.68 (95% CI 0.47–0.84, *I*^2^ = 70.31%, *P* < 0.01), respectively (Figure 2). Besides, the pooled PLR was 3.1 (95% CI 1.7–5.5, *I*^2^ = 44.55%, *P* = 0.01) and the NLR was 0.03 (95% CI 0.00–0.90, *I*^2^ = 0.00, *P* = 0.42) (Supplementary Figure 2). There was a significant difference between the good and poor responders in the DOR (pooled DOR: 103, 95% CI 4–3,003, *I*^2^ = 68.49%, *P* = 0.01) (Supplementary Figure 3). The proportion of heterogeneity was likely due to threshold effect = 1.00, demonstrating that threshold effect of the meta-analysis existed, and the differences in the cut-off values of ΔUR of ^99m^Tc-MIBI scintigraphy were related to the heterogeneity. The SROC curve showed the AUC was 0.91 (95% CI 0.88–0.93) (Figure 3).

**Figure 4:**
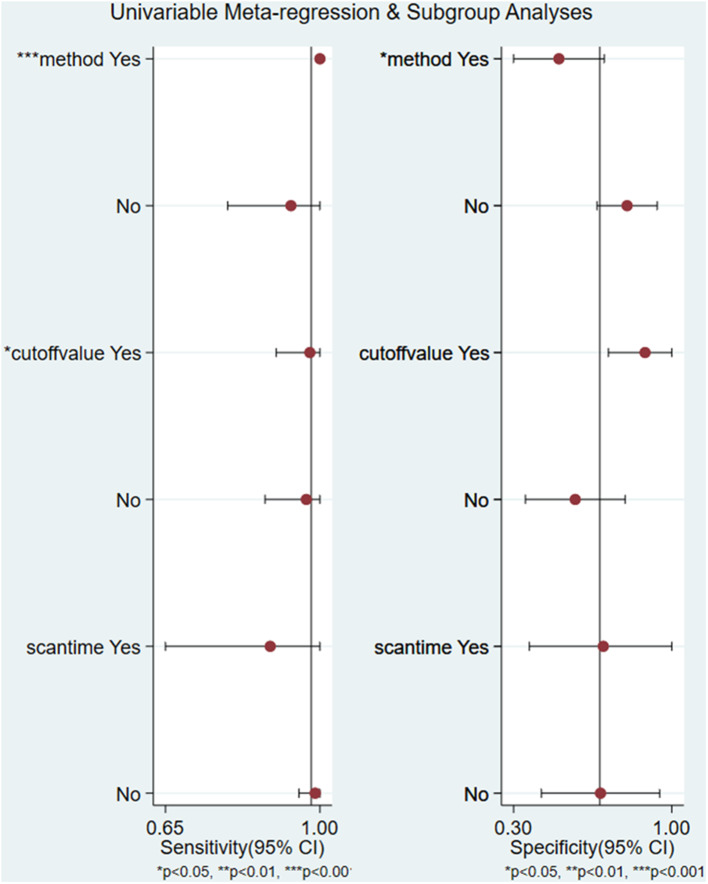
Univariate regression analysis and subgroup analysis for the uptake change ratio of ^99m^Tc-MIBI scintigraphy in preoperatively assessing the response of osteosarcoma patients to neoadjuvant chemotherapy.

Except for the parameter of NLR (*I*^2^ = 0.00, *P* = 0.42), significant heterogeneity existed among the included studies, and the overall I^2^ for bivariate model was 81% (95% CI 58–100%), *P* = 0.003. The heterogeneity of parameters including sensitivity, specificity, PLR, and DOR among the 6 studies was significant. Due to the substantial heterogeneity caused by threshold effect and non-threshold effect, univariate meta-regression analyses and subgroup analyses were performed. The results of univariate meta-regression analyses revealed that the method of retrieving data of cases of true and false positives and negatives and cut off value may be the source of heterogeneity (Figure 4). In stratified analysis based on the factors of method and cut-off value, we found that the pooled sensitivity, specificity, PLR, NLR, DOR, and AUC were 0.94 (95% CI 0.53–1.00), 0.78 (95% CI 0.50–0.92), 4.2 (95% CI 1.7–10.6), 0.08 (95% CI 0.00–0.82), 55 (95% CI 5–612), and 0.93 (95% CI 0.90–0.95), respectively in the group of obtaining data directly from the study. And the heterogeneity was *I*^2^ = 36%, *P* = 0.105, which was attenuated compared with overall heterogeneity (*I*^2^ = 81%, *P* = 0.003). In the subgroup of identifying cut-off value to minimize the number of false results, the pooled sensitivity, specificity, PLR, NLR, DOR, and AUC were 0.98 (95% CI 0.37–1.00), 0.57 (95% CI 0.34–0.77), 2.3 (95% CI 1.4–3.8), 0.04 (95% CI 0.00–2.39), 65 (95% CI 1–4327), and 0.83 (95% CI 0.80–0.86), respectively. The heterogeneity was still significant with *I*^2^ = 74% and *P* = 0.011. Owing to the limited amount of included studies in the groups of method of obtaining data indirectly from the curves and identifying ^99m^Tc-MIBI ≥30% as the cut-off value to define positive responders, pooled results could not be received in these subgroups. As mentioned above, the factors of method and cut-off value may introduce the heterogeneity, and works in consideration of the same method to retrieve data, the same criteria to identify positive responders, and the same protocol to perform ^99m^Tc-MIBI scintigraphy may help us to get an ideal conclusion in the future.

### Meta-Analysis of the Washout Rate of ^99m^Tc-MIBI for the Preoperative Assessment of Histological Response to Neoadjuvant Chemotherapy in Osteosarcoma Patients

**Figure 5:**
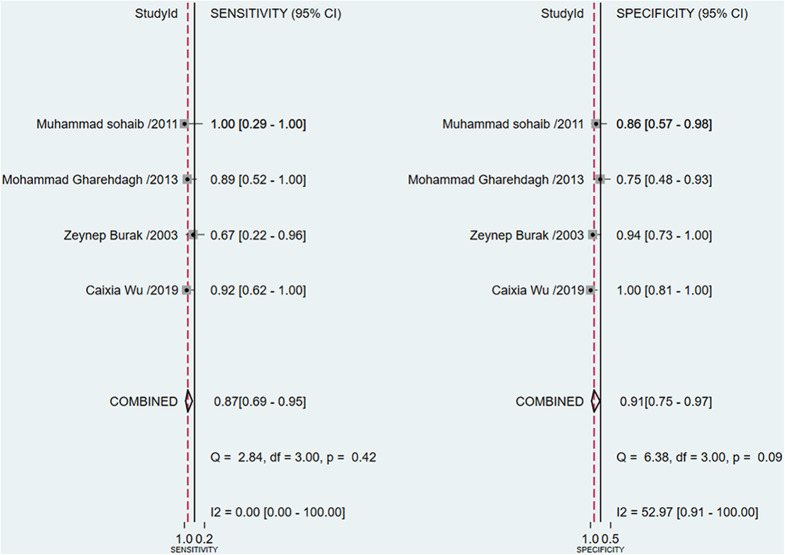
Forest plots of the pooled sensitivity and specificity for the washout rate of ^99m^Tc-MIBI scintigraphy in preoperatively assessing the response of osteosarcoma patients to neoadjuvant chemotherapy.

**Figure 6:**
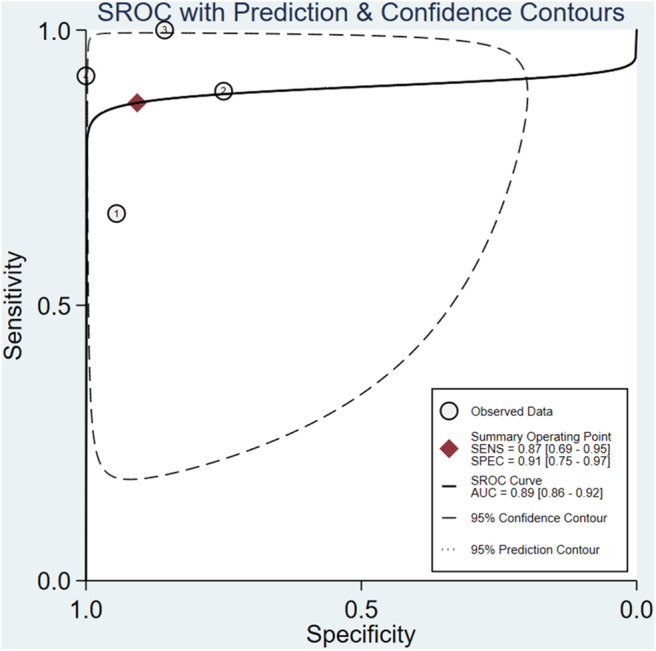
Summary receiver operating characteristic plot for the washout rate of ^99m^Tc-MIBI scintigraphy in preoperatively assessing the response of osteosarcoma patients to neoadjuvant chemotherapy.

A total of 4 studies with 96 patients were about the WR of ^99m^Tc-MIBI for the preoperative assessment of the histological response of osteosarcoma patients to neoadjuvant chemotherapy. The pooled sensitivity, specificity, PLR, NLR, and DOR were 0.87 (95% CI 0.69–0.95, *I*^2^ = 0.00, *P* =0.42), 0.91 (95% CI 0.75–0.97, *I*^2^ = 52.97%, *P* = 0.09) (Figure 5), 9.3 (95% CI 3.2–27.0, *I*^2^ = 0.00, *P* = 0.16), 0.15 (95% CI 0.06–0.37, *I*^2^ = 0.00, *P* = 0.51) (Supplementary Figure 4), and 64 (95% CI 14–301, *I*^2^ = 53.28%, *P* = 0.09) (Supplementary Figure 5), respectively. The SROC curve showed the AUC was 0.89 (95% CI 0.86–0.92) (Figure 6).

The proportion of heterogeneity was likely due to threshold effect =1.00, demonstrating that different cut-off values to define positive responders among studies may play an important role in the heterogeneity. Except for threshold effect, the overall heterogeneity for bivariate model was *I*^2^ = 0.00, *P* = 0.377. There was not significant heterogeneity among the 4 studies under the pooled sensitivity, PLR, and NLR. In terms of the pooled specificity and DOR, the heterogeneity was present with *I*^2^ = 52.97%, *P* = 0.09 and *I*^2^ = 53.28%, *P* = 0.09. However, univariate meta-regression and subgroup analyses were not performed in this part, because the number of included studies was limited, and the WR measured on pre-chemotherapy took the same criteria to define positive responders. To attenuate threshold effect and heterogeneity, more researches on the topic with a more homogenous protocol to perform ^99m^Tc-MIBI scintigraphy and criteria to define positive responders were highly needed.

### Publication Bias and Sensitivity Analysis

**Figure 7:**
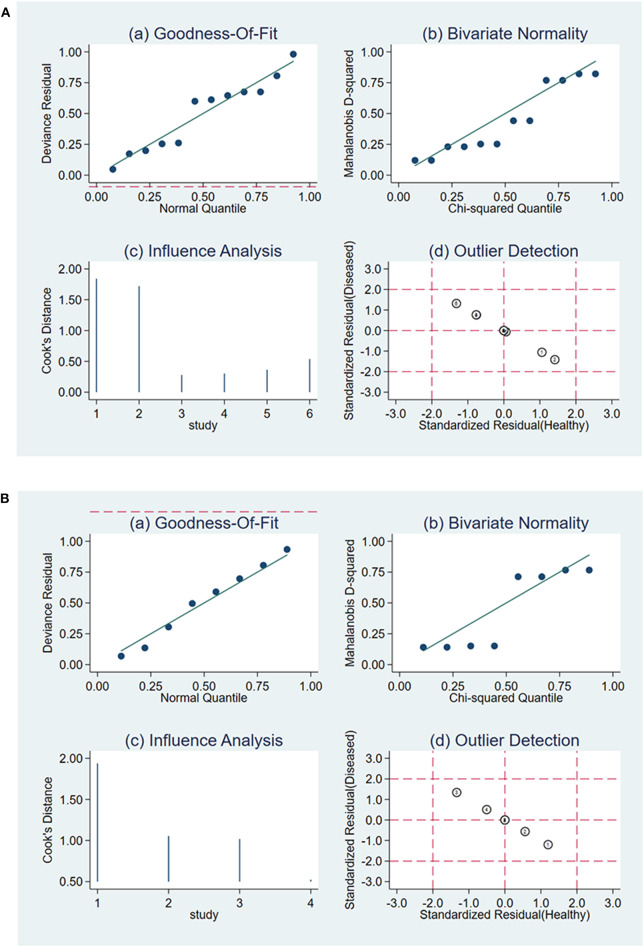
Sensitivity analysis for the uptake change ratio **(A)** and washout rate **(B)** of ^99m^Tc-MIBI scintigraphy in preoperatively assessing the response of osteosarcoma patients to neoadjuvant chemotherapy.

The Deeks' Funnel Plot hinted no publication bias in the meta-analysis of using ΔUR or WR of ^99m^Tc-MIBI to preoperatively assess the histological response of osteosarcoma patients to neoadjuvant chemotherapy (*P* = 0.74 and *P* = 0.32, respectively) (Supplementary Figure 6). The sensitivity analysis was performed, the quantile plot of residual-based goodness-of-fit and chi-squared probability plot for bivariate normality demonstrated that each included study had only minimal influence on the overall estimates, spikeplot for influence analysis and the scatterplot for checking for outliers identified no outlier studies, indicating that our meta-analysis were full of stability (Figure 7).

### Fagan Nomogram

**Figure 8:**
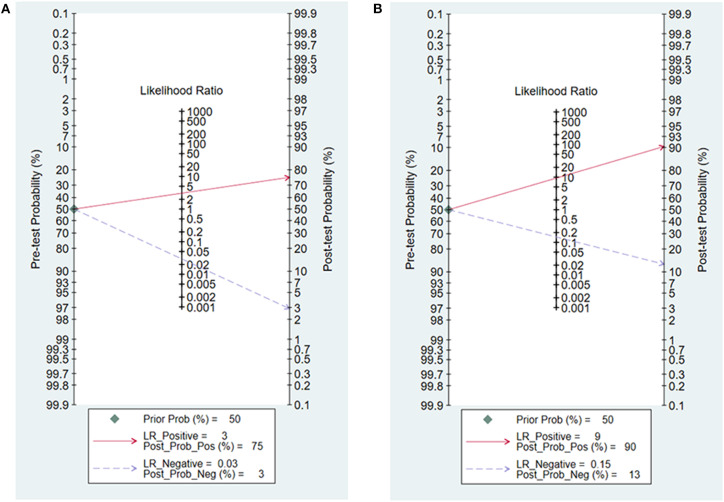
Fagan nomogram for the uptake change ratio **(A)** and washout rate **(B)** of ^99m^Tc-MIBI scintigraphy in preoperatively assessing the response of osteosarcoma patients to neoadjuvant chemotherapy.

Likelihood ratios and post-test probabilities presented in Fagan nomogram were of great value for clinicians, which provided the possibility that an osteosarcoma patient with a positive or negative ^99m^Tc-MIBI scintigraphy result was a good responder or not. In our analysis, the positive likelihood ratio of 3 implied that osteosarcoma patients defined as positive responders according to ΔUR of ^99m^Tc-MIBI were 3 times more likely to have TNR ≥ 90% than those with negative ΔUR result. Given a pretest probability of 50%, the probability for osteosarcoma patients with positive ΔUR result having TNR ≥ 90% was 75%. The negative likelihood ratio of 0.03 decreased the post-test probability to 3% for patients with negative ΔUR result, implying that the probability for an osteosarcoma patient with a negative ΔUR result having a TNR ≥ 90% was 3% (Figure 8A). According to Figure 8B, we found that the positive likelihood ratio of WR was 9, which was higher than that of ΔUR. Given a pretest probability of 50%, the post-test probability of WR was 90%. The negative likelihood ratio of 0.15 decreased the post-test probability to 13% for an osteosarcoma patient with a negative WR result, namely, the probability for osteosarcoma patients with negative WR result having TNR ≥ 90% was 13%.

## Discussion

Imaging techniques including 18F-FDG PET/CT, MRI, radionuclide imaging, etc. could be easily conducted at any treatment stage and they have played an important role in assessing the response of osteosarcoma patients to preoperative chemotherapy (14, 15). However, there has not been consensus on which imaging method is the best. Many researchers have reported that radionuclide imaging, such as thallium-201 and ^99m^Tc-MIBI scintigraphy, possessed acceptable performance in preoperatively evaluating efficacy of osteosarcoma patients to preoperative chemotherapy (29, 34). Besides, thallium-201 and ^99m^Tc-MIBI scintigraphy were relatively economical and convenient to conduct. In the aspect of physical properties, ^99m^Tc-MIBI is comparatively better than thallium-201, and the half-value periods of thallium-201 are much longer than that of ^99m^Tc-MIBI, which limits the dose and results of thallium-201 scintigraphy in poor image quality (30). ^99m^Tc-MIBI could passively diffuse through the cell membrane and accumulate in mitochondria under the effect of transmembrane potential. Metabolically active tissues containing more mitochondria will take up and retain more ^99m^Tc-MIBI (35). In osteosarcoma patients, the uptake and accumulation of ^99m^Tc-MIBI indirectly represents the presence of metabolically active osteosarcoma cells, and ^99m^Tc-MIBI scintigraphy evaluation for response to preoperative chemotherapy can be achieved by analyzing the ΔUR of tumor ^99m^Tc-MIBI before and after neoadjuvant chemotherapy. Response of osteosarcoma patients to chemotherapeutics is often significantly correlated with the level of P-gp and MRP, which can efflux chemotherapeutics out of tumor cells and thus impair the chemotherapy effect (36). ^99m^Tc-MIBI is a transport substrate of both P-gp and MRP, which would transport ^99m^Tc-MIBI out of tumor cells (37). A rapid WR of ^99m^Tc-MIBI is positively related to a high level of P-gp and MRP in tumor patients. Theoretically, response of osteosarcoma patients to chemotherapy also could be predicted by the WR of ^99m^Tc-MIBI from the early and delayed images. There have been many researches assessing the ^99m^Tc-MIBI scintigraphy for the preoperative prediction of neoadjuvant chemotherapy response in osteosarcoma patients, but the researches were limited in sample size and lack of reliability. In some researches, the conclusions were even inconsistent (32, 38). Thus, to derive more robust and reliable estimates of the evaluating performance of ^99m^Tc-MIBI scintigraphy for the response of osteosarcoma patients to neoadjuvant chemotherapy, we collected and pooled all relevant researches in this meta-analysis, which, as far as we know, had not previously been published.

The result of our analysis revealed that the proportion of heterogeneity likely due to threshold effect = 1.00, namely, threshold effect of the meta-analysis was significant, and statistical pooling of sensitivity, specificity, PLR, NLR, and DOR should be cautious. Then, the AUC serving as a global measure of test performance could be a more useful index to estimate the performance of ^99m^Tc-MIBI scintigraphy for the response of osteosarcoma patients to neoadjuvant chemotherapy. The following guidelines were accepted for interpretation of intermediate AUC values: low accuracy (0.5 ≤ AUC ≤ 0.7), moderate accuracy (0.7 ≤ AUC ≤ 0.9), and high accuracy (0.9 ≤ AUC ≤ 1) (39). According to the value of AUC, we concluded the index of ΔUR of ^99m^Tc-MIBI uptake possessed a high diagnostic accuracy. Due to the substantial heterogeneity of the meta-analysis of ΔUR of ^99m^Tc-MIBI for the preoperative assessment of the histological response of osteosarcoma patients to neoadjuvant chemotherapy, meta-regression and subgroup analyses were performed. Their results suggested that the method of retrieving data of cases of true and false positives and negatives and cut-off value may be the source of heterogeneity. As for the WR of ^99m^Tc-MIBI before chemotherapy, threshold effect existed and the heterogeneity of several parameters was insignificant except for the specificity and DOR. The index of WR of ^99m^Tc-MIBI possessed a moderate diagnostic accuracy with the value of AUC = 0.89. Owing to the limited number of included studies, meta-regression and subgroup analyses were not performed in this part. To attenuate the threshold effect and heterogeneity of our analysis, more researches on the topic with the same method to retrieve data and a more homogenous protocol to perform ^99m^Tc-MIBI scintigraphy and criteria to define positive responders were highly needed in the future. According to the value of AUC and pooled DOR, both the indexes of ΔUR and WR derived from ^99m^Tc-MIBI scintigraphy were valuable in preoperatively assessing the histological response of osteosarcoma patients to neoadjuvant chemotherapy, and ΔUR possessed a more outstanding diagnostic accuracy than that of WR. The result of sensitivity analysis identified no outlier studies and the included studies had only minimal influence on the overall estimates. Assessment of publication bias was performed through the Deeks' Funnel Plot Asymmetry Test, which did not hint any evidence of publication bias in the meta-analysis. Both the sensitivity analysis and the publication bias analysis demonstrated the result of our study was stable, reliable, and believable.

Our research was the first meta-analysis on this topic. It was based on thorough literature searches upon different electronic databases. Data extraction, study selection with well-defined inclusion and exclusion criteria, and the quality assessment of included studies using QUADAS-2 implemented in RevMan 5.3 were all performed independently by two researchers, and discrepancies were resolved through group discussion. However, several limitations of our study still should be considered. Firstly, the number of included studies and patients included in the analysis was limited, because several studies were eliminated for unavailability of getting the cases of true and false positives and negatives. More studies relevant to the topic were necessary to make our results more reliable and credible. Secondly, the threshold effect of our analysis was obvious, which would somewhat impair the credibility of our conclusions. Osteosarcoma patients with ΔUR ≥ 30% were defined as positive responders in 2 studies. However, in the other 4 studies on ΔUR, the cut-off value was identified to minimize the number of false results. Thus, original researches with a more homogenous protocol to perform ^99m^Tc-MIBI scintigraphy and criteria to define positive responders were highly needed in the future. Thirdly, heterogeneity of the meta-analysis of ΔUR of ^99m^Tc-MIBI for the preoperative assessment of histological response of osteosarcoma patients to neoadjuvant chemotherapy was significant. However, subgroup analysis was unavailable in some cases, owing to the limited number of included studies. More studies on the topic were necessary, and meta-regression and subgroup analysis based on other factors could be considered to vanish the heterogeneity. Fourthly, we did not compare the diagnostic accuracy of ^99m^Tc-MIBI scintigraphy with other imaging techniques, and we still did not reach consensus on which imaging method was most suitable to be applied in daily clinical work. Fifthly, the study design of most included studies was not clearly mentioned in text, which made the subgroup analysis based on the factor of study design could not be performed. In the end, we only included studies written in English and Chinese, which might affect our findings.

In this meta-analysis, we aimed to semi-quantitatively assess the histological response of osteosarcoma patients to neoadjuvant chemotherapy before surgery or treatment by using ΔUR or WR of ^99m^Tc-MIBI scintigraphy. According to the value of AUC and pooled DOR, both the ΔUR and WR derived from ^99m^Tc-MIBI scintigraphy were valuable in preoperatively assessing the histological response of osteosarcoma patients to neoadjuvant chemotherapy, and ΔUR possessed a more outstanding diagnostic accuracy than WR. More researches on the topic of this analysis with larger sample, the same method to retrieve data, and a more homogenous protocol to perform ^99m^Tc-MIBI scintigraphy and criteria to define positive responders were highly required to verify the outcomes. Moreover, other more accurate and convenient methods of assessing the response of osteosarcoma patients to neoadjuvant chemotherapy still need to be developed and investigated in the future.

## Data Availability Statement

All datasets generated and analyzed for this study are included in the article/Supplementary Material.

## Author Contributions

FW, ZZ, and ZS: conception and design. FW and XinH (3rd Author): literature search and identification. FW and YH: data extraction and quality assessment. FW and ZZ: data analysis. FW, SF, XiaH, XinH (6th Author), and ZS: original draft prepararion. FW and ZS: writing-review, editing and supervision. ZZ and ZS: funding acquisition.

## Conflict of Interest

The authors declare that the research was conducted in the absence of any commercial or financial relationships that could be construed as a potential conflict of interest.

## Abbreviations

TNR: tumor necrosis rate
ΔUR: uptake change ratios
WR: washout rate
P-gp: P-glycoprotein
MRP: multidrug resistance-associated protein
95% CI: 95% confidence interval
QUADAS-2: Quality Assessment of Diagnostic Accuracy Studies-2
PLR: positive likelihood
NLR: negative likelihood
DOR: diagnostic odds ratio
SROC: summary receiver operating characteristic
AUC: the area under curve.
