# Supplementary material for: 99mTc-MIBI Scintigraphy for the Preoperative Assessment of Histological Response to Neoadjuvant Chemotherapy in Patients With Osteosarcoma: A Systematic Review and a Bivariate Meta-Analysis
Source: Front Oncol. 2020 May 22;10:762. doi: 10.3389/fonc.2020.00762 (PMC7258398; doi:10.3389/fonc.2020.00762)
Supplement: Supplementary file 1 [file Data_Sheet_1.docx]

**Supplementary Materials**

**
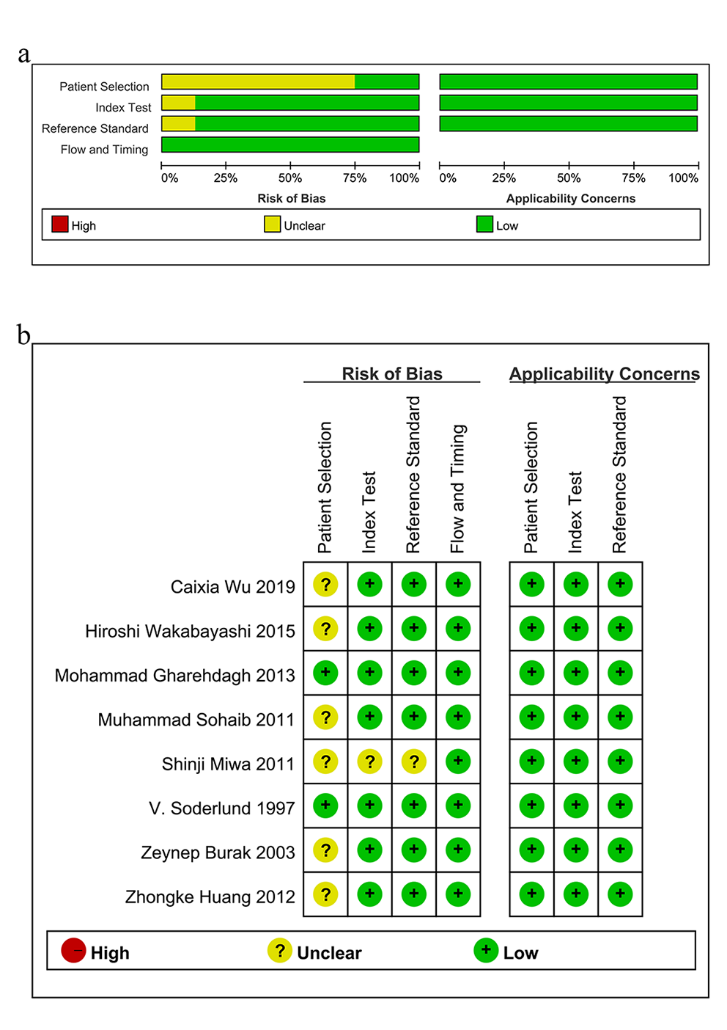
**

**Supplementary Figure 1.** Quality assessment of eligible studies (Quality Assessment of Diagnostic Accuracy Studies-2). Risk of bias and applicability concerns graph (a) and risk of bias and applicability concerns summary (b)

**
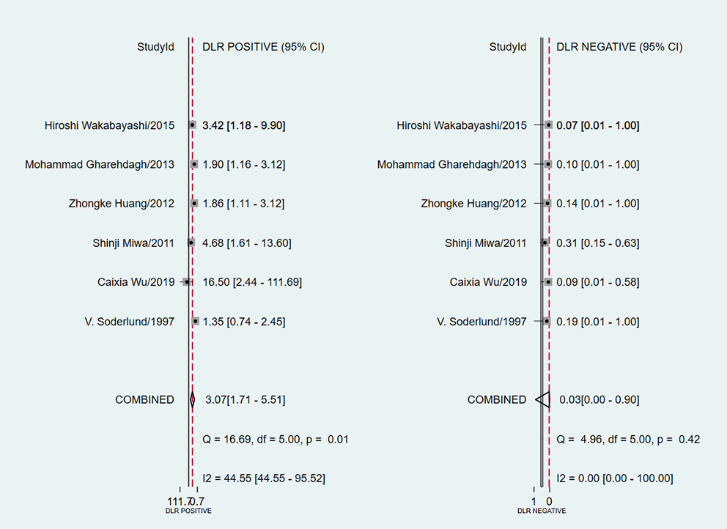
**

**Supplementary Figure 2.** Forest plots of the pooled positive and negative likelihood ratio for the uptake change ratio of ^99m^Tc-MIBI scintigraphy in preoperatively assessing the response of osteosarcoma patients to neoadjuvant chemotherapy

**
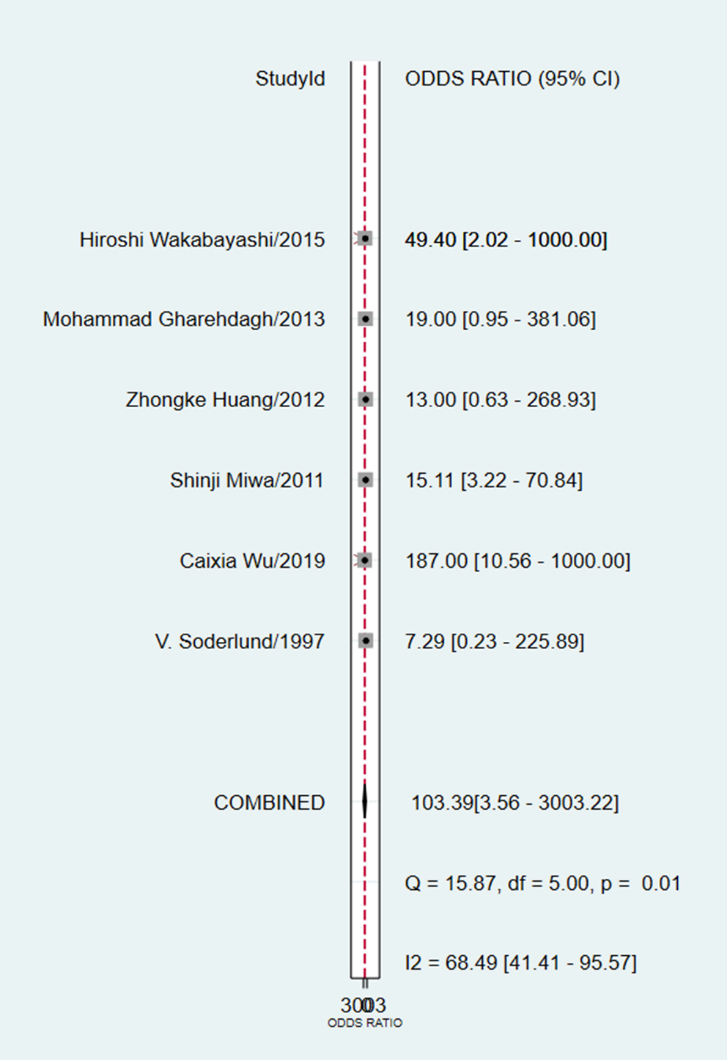
**

**Supplementary Figure 3.** Forest plots of the pooled diagnostic odds ratio for the uptake change ratio of ^99m^Tc-MIBI scintigraphy in preoperatively assessing the response of osteosarcoma patients to neoadjuvant chemotherapy

**
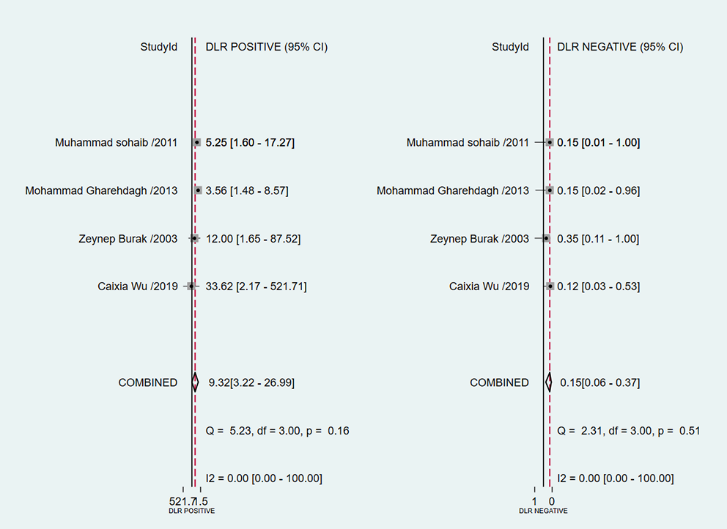
**

**Supplementary Figure 4.** Forest plots of the pooled positive and negative likelihood ratio for the washout rate of ^99m^Tc-MIBI scintigraphy in preoperatively assessing the response of osteosarcoma patients to neoadjuvant chemotherapy

**
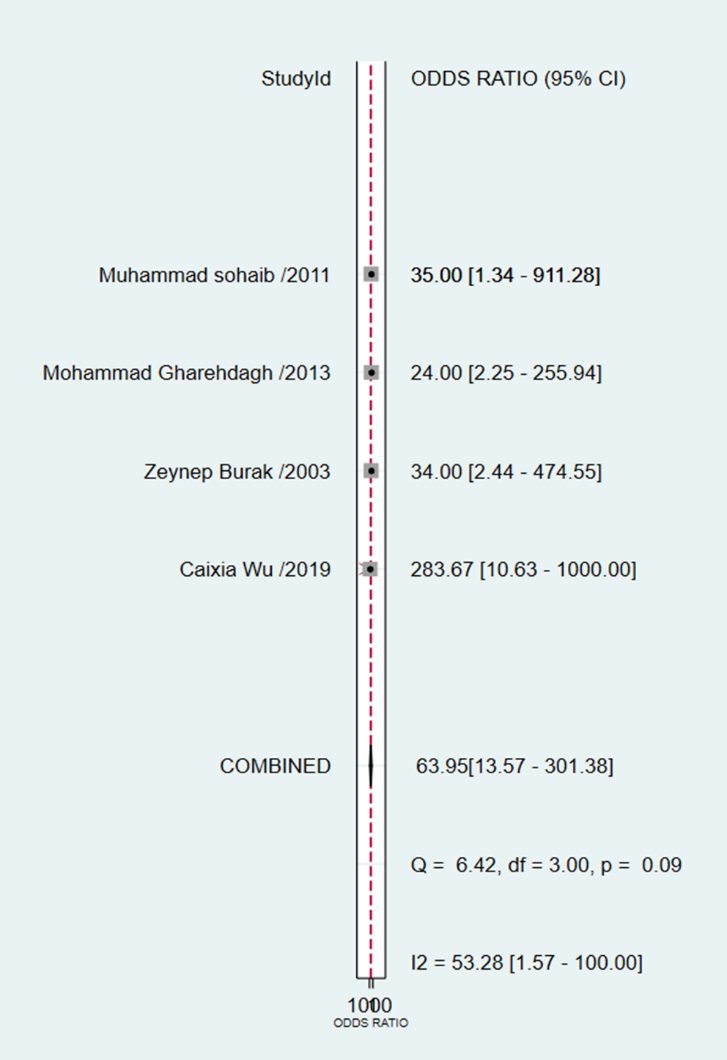
**

**Supplementary Figure 5.** Forest plots of the pooled diagnostic odds ratio for the washout rate of ^99m^Tc-MIBI scintigraphy in preoperatively assessing the response of osteosarcoma patients to neoadjuvant chemotherapy

**
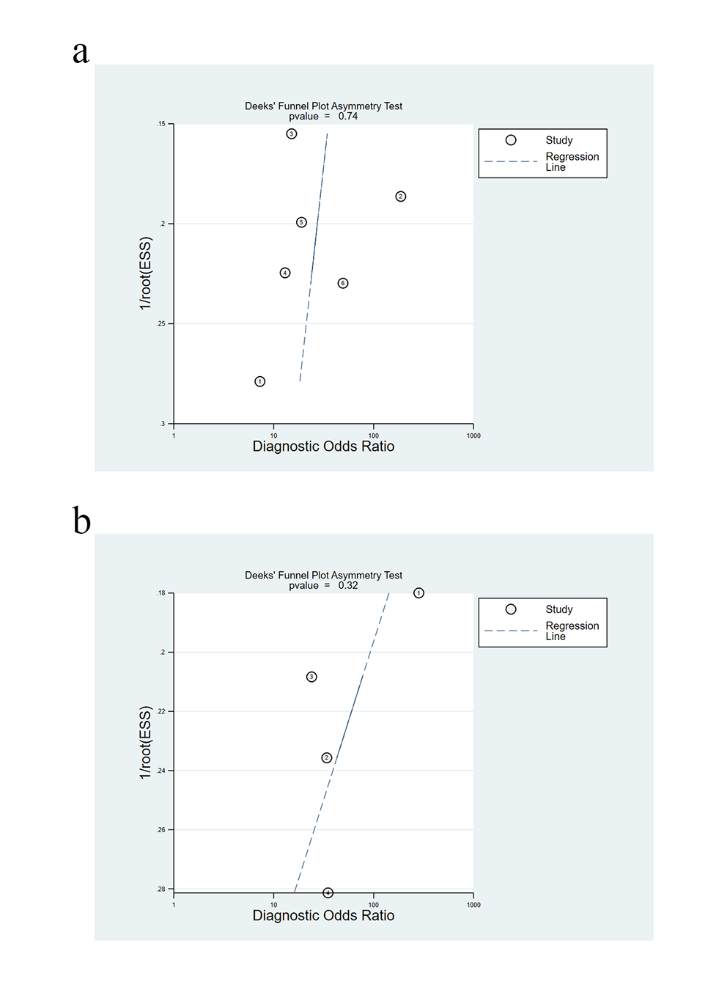
**

**Supplementary Figure 6.** Deeks’ Funnel Plot Asymmetry Test for the uptake change ratio (a) and washout rate (b) of 99mTc-MIBI scintigraphy in preoperatively assessing the response of osteosarcoma patients to neoadjuvant chemotherapy
